# Aβ profiles generated by Alzheimer’s disease causing *PSEN1* variants determine the pathogenicity of the mutation and predict age at disease onset

**DOI:** 10.1038/s41380-022-01518-6

**Published:** 2022-04-01

**Authors:** Dieter Petit, Sara Gutiérrez Fernández, Katarzyna Marta Zoltowska, Thomas Enzlein, Natalie S. Ryan, Antoinette O’Connor, Maria Szaruga, Elizabeth Hill, Rik Vandenberghe, Nick C. Fox, Lucía Chávez-Gutiérrez

**Affiliations:** 1 VIB-KU Leuven Center for Brain & Disease Research, Herestraat 49 box 602, 3000 Leuven, Belgium; 2 Department of Neurosciences, Leuven Brain Institute, KU Leuven, Herestraat 49 box 602, 3000 Leuven, Belgium; 3 Center for Mass Spectrometry and Optical Spectroscopy (CeMOS), Mannheim University of Applied Sciences, Paul-Wittsack Str. 10, 68163 Mannheim, Germany; 4 UK Dementia Research Institute at UCL, Queen Square, WC1N 3BG London, UK; 5 Dementia Research Centre, Department of Neurodegenerative Disease, UCL Queen Square Institute of Neurology, Queen Square, WC1N 3BG London, UK; 6 Laboratory for Cognitive Neurology, Department of Neurosciences, KU Leuven, Herestraat 49 box 1027, 3000 Leuven, Belgium; 7 Neurology Department, University Hospitals Leuven, Herestraat 49, 3000 Leuven, Belgium

**Keywords:** Molecular biology, Neuroscience, Biochemistry

## Abstract

Familial Alzheimer’s disease (FAD), caused by mutations in Presenilin *(PSEN1/2)* and Amyloid Precursor Protein *(APP*) genes, is associated with an early age at onset (AAO) of symptoms. AAO is relatively consistent within families and between carriers of the same mutations, but differs markedly between individuals carrying different mutations. Gaining a mechanistic understanding of why certain mutations manifest several decades earlier than others is extremely important in elucidating the foundations of pathogenesis and AAO. Pathogenic mutations affect the protease (PSEN/γ-secretase) and the substrate (APP) that generate amyloid β (Aβ) peptides. Altered Aβ metabolism has long been associated with AD pathogenesis, with absolute or relative increases in Aβ42 levels most commonly implicated in the disease development. However, analyses addressing the relationships between these Aβ42 increments and AAO are inconsistent. Here, we investigated this central aspect of AD pathophysiology via comprehensive analysis of 25 FAD-linked Aβ profiles. Hypothesis- and data-driven approaches demonstrate linear correlations between mutation-driven alterations in Aβ profiles and AAO. In addition, our studies show that the Aβ (37 + 38 + 40) / (42 + 43) ratio offers predictive value in the assessment of ‘unclear’ *PSEN1* variants. Of note, the analysis of PSEN1 variants presenting additionally with spastic paraparesis, indicates that a different mechanism underlies the aetiology of this distinct clinical phenotype. This study thus delivers valuable assays for fundamental, clinical and genetic research as well as supports therapeutic interventions aimed at shifting Aβ profiles towards shorter Aβ peptides.

## Introduction

Alzheimer’s disease (AD) is characterised neuropathologically by the accumulation of extracellular cerebral amyloid plaques, composed of aggregated amyloid β (Aβ) peptides, intracellular neurofibrillary tangles of hyperphosphorylated aggregated tau, reactive micro/astroglia, dystrophic neurites and progressive neuronal loss. At the clinical level, the disease manifests with progressive cognitive and functional decline that devastates the lives of AD patients, their families and caregivers [1]. Genetic analyses, in vitro and in vivo biochemical data, together with longitudinal imaging studies strongly support the notion that altered Aβ production and/or clearance, resulting in Aβ build-up in the brain, trigger pathogenic cascades leading to AD [2]. In most cases AD is sporadic (SAD) with a late age at disease onset (AAO > 65 years). However, in rare cases the disease is associated with autosomal dominant inheritance and typically manifests much earlier (AAO: 24–60 years) [3].

More than 300 pathogenic mutations in presenilin 1 or 2 (*PSEN1/PSEN2*) and amyloid precursor protein (*APP*) genes have been identified in these autosomal dominant, familial AD (FAD) cases, providing a unique, genetically validated model to study AD pathogenesis. Notably, the affected genes are functionally related: encoding the substrate (APP) and the catalytic subunit (PSEN1) of the γ-secretase protease (GSEC) involved in the generation of Aβ peptides. The AAO of clinical symptoms is relatively consistent within families and between carriers of the same FAD-linked mutations, but differs markedly between mutations. Carriers of pathogenic *PSEN1* variants may present symptoms as early as 24 years of age or later into their 60 s [3]. The molecular and mechanistic foundations of why some mutations manifest symptomatically decades earlier than others are of great importance in understanding AD pathogenesis. This is also of practical importance for family members, since this information can help to clarify pathogenicity of novel mutations or to better predict AAO in mutation carriers.

**Fig. 1:**
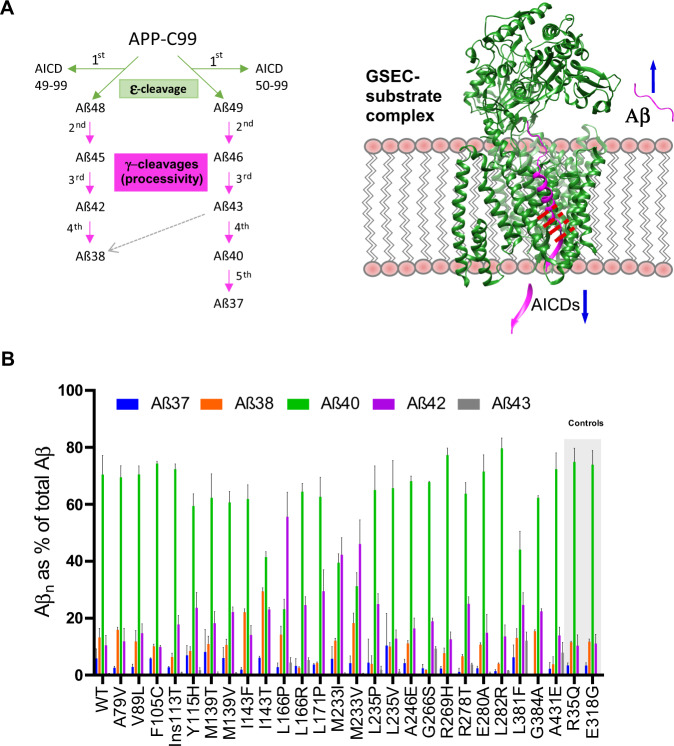
Molecular composition of secreted Aβ profiles derived from pathogenic PSEN1/GSEC variants. **A** (Left panel) GSEC co-structure with APP_C83_ (PDB: 6IYC). (Right panel) Current model of APP_C99_ cleavage by GSEC. In the first step, endopeptidase (ε-) cleavage of APP_C99_ by GSEC generates the APP intracellular domain (AICD_50–99_ or AICD_49–99_) and a de novo Aβ substrate (Aβ48 or Aβ49, respectively). While AICDs are released into the cytosol, the membrane bound Aβ fragments are further processed in a sequential manner through γ-cleavages. **B** Aβ profiles (relative abundance of the Aβ37, Aβ38, Aβ40, Aβ42 and Aβ43 peptides with respect to the total Aβ levels (Aβ37 + 38 + 40 + 42 + 43)) generated by wild type or mutant PSEN/GSECs. Data is presented as mean ± SD, *N* ≥ 4 independent experiments (see also Table S2).

GSECs are multimeric intramembrane proteases, with PSEN1 or PSEN2 [4–6], nicastrin (NCSTN) [7], presenilin enhancer 2 (PEN-2) [8] and anterior pharynx defective 1 A or B (APH1A or B) [8, 9] as essential components (Fig. 1A). The GSEC-mediated proteolysis of APP_C99_ generates a mixture of Aβ peptides of various lengths (predominantly 37–43 amino acids) by a rather unique sequential proteolytic mechanism [10]. Pathogenic mutations alter the proportions of the different Aβ peptides (Aβ profiles) that are generated by GSECs, and the absolute and/or relative (to Aβ40) increments in Aβ42 levels have long been considered a hallmark of pathogenic mutations [11–20]. Increases in Aβ42 production are supported by in vivo stable isotope labelling kinetic (SILK) studies comparing *PSEN1* mutation carriers with non-carriers [21]. Nevertheless, several studies have come to opposite conclusions [17, 18, 22–24] and raised questions over the pathogenic role of Aβ in AD pathogenesis. It is important to mention that all these studies focused on the levels of just two Aβ peptides (Aβ40 and Aβ42), out of the complex mixture of Aβ species generated (Aβ37, Aβ38, Aβ40, Aβ42 and Aβ43) despite previous analyses demonstrating that the generation of other Aβ peptides (other than Aβ42 and Aβ40) is also significantly affected in FAD conditions [25–30]. Therefore, crucial questions regarding the impact of pathogenic mutations on Aβ profile composition and their connection with disease severity remain open.

Indeed, the relationship between molecular aspects of FAD-causing mutations and clinical phenotypes constitutes a fundamental, unresolved question with implications for basic and clinical research, as well as for therapeutic development. In this regard, our previous studies have demonstrated that FAD-linked mutations in PSEN1 share a common mechanism: they destabilize GSEC-APP/Aβ_n_ (Enzyme-Substrate, E-S) interactions during the sequential proteolysis, and thereby promote the premature release of longer Aβ_n_ peptides [29].

Here, we hypothesized that the analysis of the full spectrum of Aβ profiles (including Aβ37, Aβ38, Aβ40, Aβ42 and Aβ43) better reflects mutation pathogenicity than the simpler but widely used Aβ 42/40 ratio.

We therefore performed comprehensive (as possible) analysis of Aβ profiles generated by 25 mutant PSEN1/GSECs that span a wide range of AAOs. To investigate potential relationships between the molecular composition of FAD-linked Aβ profiles and disease severity (reflected by AAO), we performed mechanism- and data-driven analyses. Both demonstrate that the mutation-mediated alterations in Aβ profiles correlate linearly with AAO, and significantly, the linear correlation established here has predictive value in the assessment of *PSEN1* variants of unclear pathogenicity, limited family history or heterogenous/complex clinical phenotypes.

Collectively, these analyses strongly support the critical importance of alterations in the relative amounts of Aβ peptides in FAD pathogenesis, while the derived novel insights help to clarify the molecular determinants modulating AAO and encourage drug discovery efforts targeting Aβ production to promote the generation of shorter peptides. Furthermore, they provide tools for quantitative estimation of the effects of Aβ profile alterations on AAO, which may be clinically useful for families where historic estimates of AAO are uncertain. Finally, these tools may facilitate the discovery of genetic modifiers of AAO by identifying FAD *PSEN1* mutation carriers presenting a mismatch between the biochemically estimated and clinical AD onsets.

## Results

### Changes in Aβ profile composition -linked to pathogenic *PSEN1* variants- correlate with AAO

We investigated the molecular composition of Aβ profiles generated by 25 mutant PSEN1/GSECs associated with a broad range of AAOs (24–60 years, Table 1). These substitutions are distributed throughout the PSEN1 3D structure; apart from five paired mutations that occur at the same positions but are associated with widely differing AAOs (M139T/V, I143F/T, L166P/R, M233I/V and L235P/V). As non-pathogenic controls, we used E318G and R35Q variants; both derived from a genome aggregation database (>300,000 individuals, GnomAD https://gnomad.broadinstitute.org/) search.

**Table 1:** Studied FAD-linked PSEN1 mutations, their location in PSEN1/GSEC and reported ages at AD onset (AAOs).

PSEN1 mutation	Position	Mean AAO (range)	# in Fig. 2B
L166P	TMD 3	23.5 (23–24)	1
M233V	TMD 5	24.8 (15–34)	2
M233I	TMD 5	27 (24–30)	3
L381F	TMD 7	29.8 (28–32)	4
I143T	TMD 2	31.9 (28–38)	5
L235P	TMD 5	32.5 (29–39)	6
G384A	TMD 7	36.0 (26–45)	7
R278T	TMD 6	37.0	8
Y115H	Loop 1	37.0 (30–47)	9
L166R	TMD 3	37.3 (32–44)	10
L171P	TMD 3	38 (36–40)	11
A431E	IC loop	39.4 (36–53)	12
M139V	TMD 2	39.9 (32–48)	13
Ins113T (intron 4)	Loop 1	42.1 (35–45)	14
L282R	IC loop	43.8 (35–50)	15
G266S	TMD 6	45.0 (33–45)	16
M139T	TMD 2	47.3 (39–51)	17
L235V	TMD 5	47.4 (44–59)	18
E280A	IC loop	48.1 (46–52)	19
F105C	Loop 1	48.6 (45–51)	20
V89L	TMD 1	48.7 (38–51)	21
A246E	TMD 6	49.1 (40–66)	22
I143F	TMD 2	55.0 (51–59)	23
R269H	TMD 6	56.4 (50–62)	24
A79V	TMD 1	60.6 (53–78)	25
R35Q	N-term	—	—
E318G	IC loop	—	—

AAOs were defined accordingly to the Alzforum *PSEN1* mutation database (https://www.alzforum.org/mutations/psen-1), AD&FTD mutation database from the University of Antwerp (https://www.molgen.ua.ac.be/admutations) and the available literature [65]. R35Q and E318G substitutions were selected as non-pathogenic.

*Ins113T* p. L113-I114InsT, *TMD* transmembrane domain, *Loop 1* extracellullar loop between TMD1-2, *IC loop* intracellular loop between TMD 6-7, *N-term* N-terminal region.

We generated PSEN1 wild type and mutant cell lines on a *Psen* null background and verified that the expression of human PSEN1 efficiently reconstituted GSEC, as indicated by the restored levels of mature NCSTN, PEN-2 and PSEN1 C-and N-terminal fragments (Fig. S1). To determine the effects of the PSEN1 variants on the Aβ production, cells were transduced with human APP_C99_-expressing adenoviruses and the levels of secreted Aβ37, 38, 40, 42 and 43 peptides quantified. The analysis demonstrated an enrichment, albeit to different extents, in the abundance of longer Aβ species, relative to the shorter ones in the FAD-linked GSEC generated Aβ profiles, compared to the wild type reference (Fig. 1B). In contrast, Aβ profiles for the R35Q, E318G and wild type cell lines were virtually identical, supporting the non-pathogenic nature of these substitutions.

**Fig. 2:**
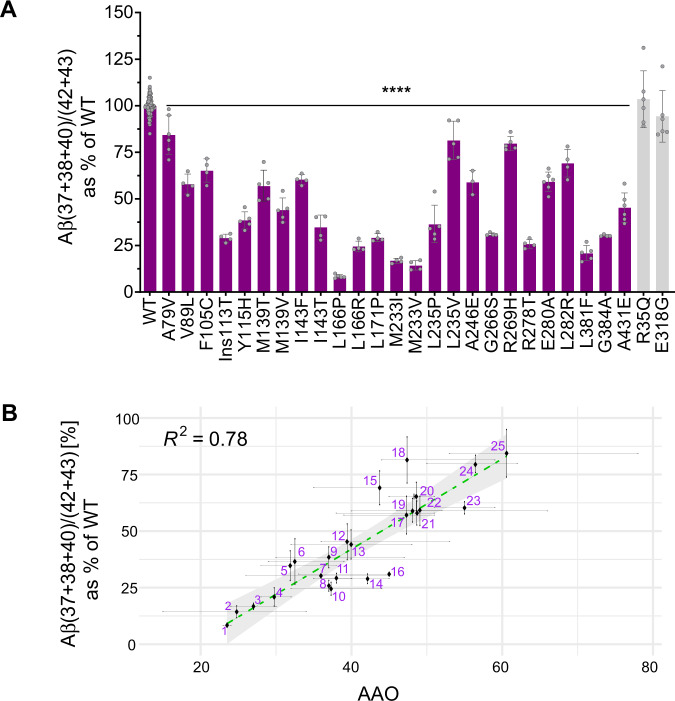
Changes in Aβ profile composition -linked to pathogenic PSEN1 mutants- correlate linearly with AAO. **A** The efficiency of the 4^th^ enzymatic turnover of APP_C99_ quantified by the Aβ (37 + 38 + 40) / (42 + 43) ratio. Data are represented as mean ± SD, *N* ≥ 4 independent experiments. One-way ANOVA followed by Dunnett’s post-hoc test with comparison to wild type was used to determine statistical significance (*p* < 0.05); ****p < 0.0001, (F(DFn, DFd): F (27, 185) = 200.6); (**B**) Correlative analysis between AAO and Aβ (37 + 38 + 40) / (42 + 43) ratio (efficiency of the 4^th^ catalytic turn-over) (Y = 1.996*X - 37.8). The 95% confidence interval (light grey surface) and correlation coefficient (R^2^) are shown. The error bars present SD and range for Aβ ratio and AAO, respectively.

To quantify GSEC processivity, we used the Aβ (37 + 38 + 40) / (42 + 43) ratio, which weights the levels of the products by the levels of the substrates of the 4^th^ catalytic turnover (APP_C99_→Aβ49→46→**43→40**→**37** and APP_C99_→Aβ48→45→**42**→**38**), and thus provides an overall measure of GSEC processivity along both product lines (Fig. 1A). Note that the sum of Aβ37 and Aβ40 represents the total Aβ40 product generated, as it includes both released as well as converted (to Aβ37) Aβ40. In contrast to the control PSEN1 variants, FAD-linked PSEN1 mutants consistently impair the efficiency of the sequential γ-cleavages (GSEC processivity) (Fig. 2A), which is in line with previous studies [25, 31]. Previous assessment of GSEC processivity, showing consistent decrements in the Aβ 38/42 and Aβ 37/40 ratios across pathogenic *PSEN1* variants [25], did not test the connection with AAO. However, our analyses of the thermostabilities of GSEC-APP/Aβ_n_ interactions did reveal a linear correlation between the degree of mutation-driven destabilization and AAO, implicating longer Aβ peptides in pathogenesis [29]. Here, we went a step further and tested the hypothesis that altered Aβ profile composition, arising from mutation-driven destabilization of E-S, determines AAO.

We evaluated the relationships between FAD-linked changes in Aβ profiles and AAO (Fig. 2B). Strikingly, the results revealed a significant linear correlation between the Aβ (37 + 38 + 40) / (42 + 43) ratio and AAO (R^2^ = 0.78, *P* < 0.0001). This indicates that mutation-driven alterations in full Aβ profiles not only trigger AD but importantly largely determine AAO. Conceptually, these findings provide further support to the ‘GSEC metastability model’ which proposes that intrinsic (mutations) and/or extrinsic (environmental) factors modulating GSEC-APP/Aβ_n_ interactions alter the risk for AD [29].

### The simplified Aβ 40/42 ratio serves as a surrogate in the assessment of AAO

Given the long-standing precept in the field that AD pathogenesis is closely linked to increments in Aβ42 levels, we also assessed the relationships between potential mechanisms leading to changes in Aβ42 levels and AAO. According to the current model for the GSEC-mediated cleavage of APP_C99_ [10, 32, 33], relative elevations in Aβ42 may arise either from changes in the GSEC product line preference (favouring the APP_C99_→Aβ48→45→42→38 line over the APP_C99_→Aβ49→46→43→40→37 line) and/or selective impairment in the cleavage of Aβ42 to Aβ38 (Fig. 1A). Accordingly, we calculated the Aβ (38 + 42) / (37 + 40 + 43) and Aβ 38/42 ratios and plotted them against AAO. The correlation coefficients were R^2^ = 0.42 (*P* < 0.001) (Fig. S2A) and R^2^ = 0.45 (*P* < 0.001) (Fig. S2B), respectively. FAD-linked PSEN1 variants lower the Aβ 38/42 ratio and may favour the Aβ42 product line [25]; however, these results demonstrate that their impairing effects on these GSEC features do not strongly correlate with clinical onset.

We then considered whether changes in GSEC processivity would lead to changes in Aβ42 levels relative to Aβ40 [25]. The assessment of the relationship between the Aβ 40/42 ratio and AAO revealed a correlation factor of R^2^ = 0.72 (*P* < 0.0001) (Fig. S2C).

Intriguingly, the analysis of the widely used Aβ 42/40 ratio versus AAO revealed a relatively weak, although significant, correlation (R^2^ = 0.54, *P* < 0.0001) (Fig. S2D). Furthermore, statistical analysis using the ROUT test marked the L166P, M233I and M233V mutations (1, 2 and 3 in Fig. S2D, respectively), as outliers. The statistical analysis suggests that these are ‘non-representative’ FAD mutations that exert pathogenicity via a distinct mechanism. Our previous thermostability analysis however has proven the destabilizing nature of the L166P mutation, supporting a common pathogenic mechanism. The very high Aβ 42/40 ratios for the L166P and M233V are driven by the drastic decrements in Aβ40 and large increments in Aβ42 levels.

Collectively, the mechanism-driven analyses of Aβ profiles strongly support the pathogenic role of Aβ, highlight the linear relationship between GSEC processivity and AAO, and propose the Aβ 40/42 ratio as a simplified measurement in the assessment of AAO for *PSEN1* variants.

### Data-driven analysis demonstrates a strong correlation between longer Aβ peptides and AAO

**Fig. 3:**
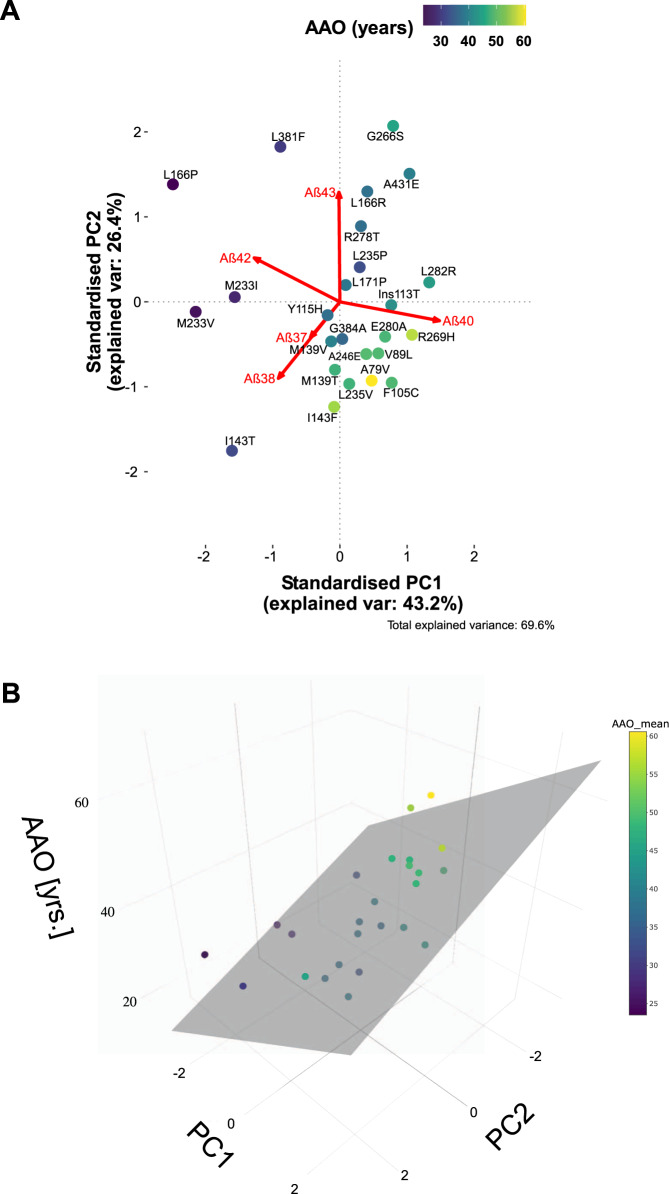
Principal component analysis of the Aβ profiles vs AAO. **A** PCA biplot demonstrating the contribution of changes in the generation of particular Aβ species to the mutation severity (AAO). See also Fig. S3 for further information about the PCA. **B** Multivariate linear model based on the data-driven PCA analysis to investigate the correlation with AAO.

We also performed an unbiased, data-driven analysis to further investigate the underlying determinants of AAO. We applied a principal component analysis (PCA) to project the multidimensional dataset (5 variables: Aβ37, 38, 40, 42 and 43) into a new coordinate system according to variance. The first two principal components (PC1 and PC2), explaining 69.6% of the total variance in the data (Fig. S3), were selected for the analysis and visualisation (Fig. 3A). The PCA biplot shows that Aβ40 and Aβ42 have antagonistic roles in PC1, while PC2 is negatively influenced by the highly related Aβ37 and Aβ38 and positively influenced by Aβ43. Colour coding, according to the AAO, revealed that the mutations associated with earlier AAO populate the quadrant II (−/+) and oppose the late onset variants in quadrant IV (+/−). The results imply positive associations between (relatively) high Aβ42 and earlier onset. In addition, the antagonistic Aβ40 and Aβ42 roles suggest that changes in the product line preference of GSEC influences AAO. We note that shifting product line in favour of Aβ42 and Aβ38 production is a feature of many *APP* mutations [25, 34].

Next, we used the PCA and the linked AAO data to build a multivariate linear model (Fig. 3B), the adjusted R² for which equalled to 0.7. The unbiased PCA thus supports a strong correlation between changes in Aβ profiles and AAO, and links longer Aβ forms with earlier clinical onsets.

### Aβ profile analysis allows prediction of the AAO for novel and ambiguous *PSEN1* variants

Advances in next generation sequencing will lead to the wider application of genetic testing and consequent discovery of variants of unknown pathogenic significance. Although a number of algorithms help to predict mutation pathogenicity, the biochemical assessment of pathogenicity and the estimation of a likely AAO in carriers of novel *PSEN1* variants is of high relevance in the clinical setting, especially in cases where there is an unclear or censored family history or a de novo mutation.

The strong correlation between the Aβ (37 + 38 + 40) / (42 + 43) ratio and the AAO motivated us to test its predictive value. For this, we selected three established FAD-linked *PSEN1* mutations (V142I, V393F and P433S), one variant of unclear pathogenicity (S132A) [3] and two novel FAD-linked *PSEN1* mutations (G266C and L282P) [35]. The S132A variant has been classified as likely deleterious and predicted to be “probably damaging” by Polyphen but “neutral” by Provean.

In addition, we evaluated two FAD-causing *PSEN1* variants (Y154N and T291P) where motor symptoms (spastic paraplegia, SP) precede cognitive decline by several years [36, 37]. For these cases, we asked whether the Aβ (37 + 38 + 40) / (42 + 43) ratio predicts clinical AD or SP onset (or neither). Finally, two mutations in *PSEN1* previously -and contentiously- associated with frontotemporal dementia (FTD) (L113P and V412I) were also tested.

**Fig. 4:**
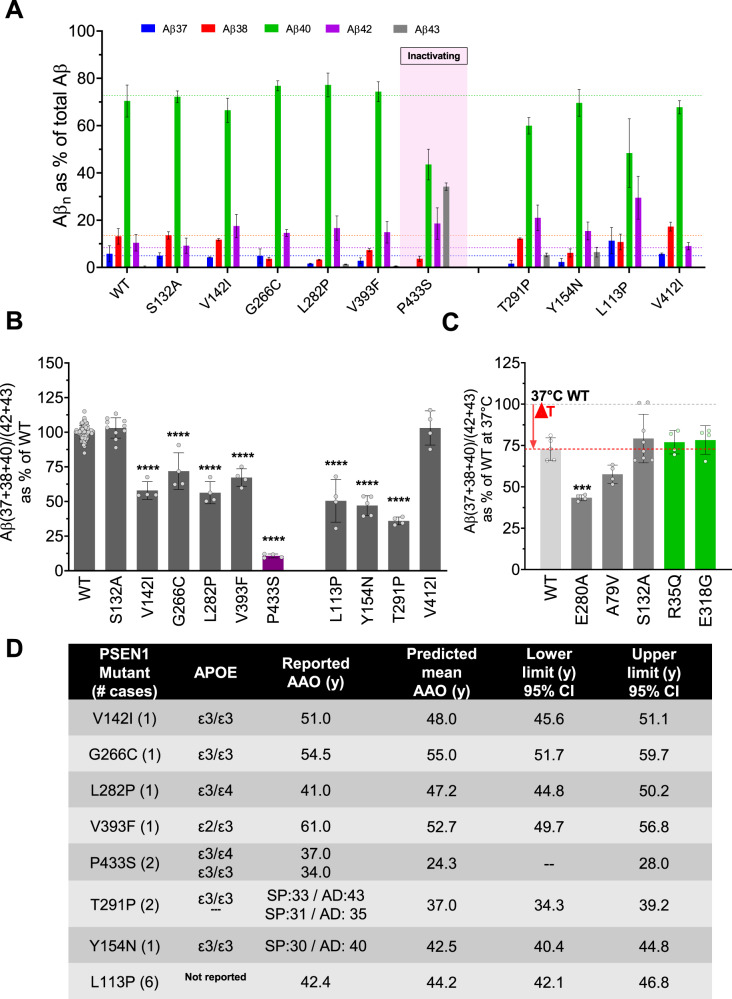
The AAO for novel, unclear or ambiguous PSEN1 variants can be estimated by Aβ profiles. **A** Secreted Aβ profiles normalised to total Aβ (defined as the sum of measure Aβ peptides). **B** Efficiency of the 4th GSEC turnover quantified by the Aβ (37 + 38 + 40) / (42 + 43) ratio. One-way ANOVA followed by Dunnett’s post-hoc test with comparison to wild type was used to determine statistical significance (*p* < 0.05); ****p < 0.0001, (F(DFn, DFd): F(10, 124) = 191.1. **C** Cell-based GSEC thermoactivity assays (42 °C for 24 h, relative to 37 °C) enable assessment of the destabilizing nature of *PSEN1* variants. The Aβ (37 + 38 + 40) / (42 + 43) ratio shows that the elevated temperature reduces GSEC processivity of the wild type and mutant protease complexes (ΔT), with further additive effects seen for destabilizing variants. One-way ANOVA followed by Dunnett’s post hoc test in comparison with wild type was used to determine the statistical significance (*p* < 0.05); ***p < 0.0001 compared to wild type at 37 °C; (F(DFn, DFd): F(5, 23) = 9.834. Aβ profiles generated in cell-based thermoactivity assays by different cell lines are shown in Fig. S4B. The data are shown as mean ± SD, *N* ≥ 4 independent experiments. **D** Table presents the estimated AAO ± 95% CI (lower and upper limits) for the indicated FAD-linked PSEN1 mutations.

Employing analogous cell-based assays as in the previous analysis, we assessed the effects of the mutations on Aβ profiles. Prior to Aβ analysis, we checked the reconstitution of active GSECs in the mutant cell lines by western blotting. All tested mutant PSEN1s reconstituted mature and active GSEC complexes, with the exception of the P433S variant, which exhibited reduced PSEN1 endoproteolysis (Fig. S1). Accordingly, the P433S cell line produced substantially lower total Aβ (Fig. S4A) but enriched for Aβ43 (Fig. 4A).

We next calculated the Aβ (37 + 38 + 40) / (42 + 43) ratios for the tested mutants and estimated the ‘intrinsic’ (biochemical) AAOs by interpolation analysis, using the equation derived from Fig. 2B. From the FAD test cohort, the GSEC/PSEN1 S132A mutant generated Aβ profiles identical to wild type GSEC, suggesting that the substitution is not pathogenic (Fig. 4A, B). To investigate this further, we tested whether the S132A mutation exerts any destabilizing effects on GSEC-APP/Aβ_n_ interactions by performing cell-based thermoactivity assays. We have previously shown that elevated temperature acts synergistically with the destabilizing effects of pathogenic *PSEN1* mutations; hence the thermoactivity assays enable uncovering of subtle destabilizing effects associated with mildly pathogenic *PSEN1* variants [29, 38]. As expected, elevated temperature lowered GSEC processivity and shifted Aβ profiles towards the longer forms in all tested cell lines (Fig. 4C and S4B). No significant differences between the control and the S132A mutant cell lines were observed, demonstrating the non-destabilizing nature of this variant.

Comparison of the predicted AAOs for the other tested FAD-linked substitutions with the clinical data showed that the actual AAOs for the V142I, G266C and L282P cases were within 3, 0.5 and ~6 years, respectively, of the predicted ones, while the AAOs for the V393F and P433S variants differed from the estimated AAO by ~8 and ~12 years (Fig. 4D). Of relevance, the V393F case carried the APOE ε2 genotype, shown to modulate AD risk and onset [39]. With regard to the P433S, a plausible explanation for the observed mismatch could be connected to its significant GSEC inactivating effects (Fig. S4A), similarly to previously proposed pathogenic mechanism of the R278I mutation [29] (see discussion).

For the T291P and Y154N variants, the estimated biochemical ages at onset overlap with the onset of AD, rather than with the one observed for the SP phenotype (Fig. 4D). This suggests that altered processing of APP underlies the cognitive changes, while the (earlier) motor symptom phenotype may potentially arise from altered GSEC-mediated processing of another, yet to be determined, GSEC substrate(s).

Finally, the Aβ profile analysis of the FTD-linked PSEN1 mutants demonstrated no significant changes for the V412I variant, relative to the wild type (Fig. 4D). In contrast, the analysis of the mutant L113P cell line revealed significant changes in Aβ profiles (Fig. 4A) that translated into reduced Aβ (37 + 38 + 40)/(42 + 43) ratio (Fig. 4B). Interpolation analysis predicted an AAO of 44.2 years (Fig. 4D).

### Aβ profile analysis reveals mechanistic similarity of PSEN1 and PSEN2 type GSEC complexes

**Fig. 5:**
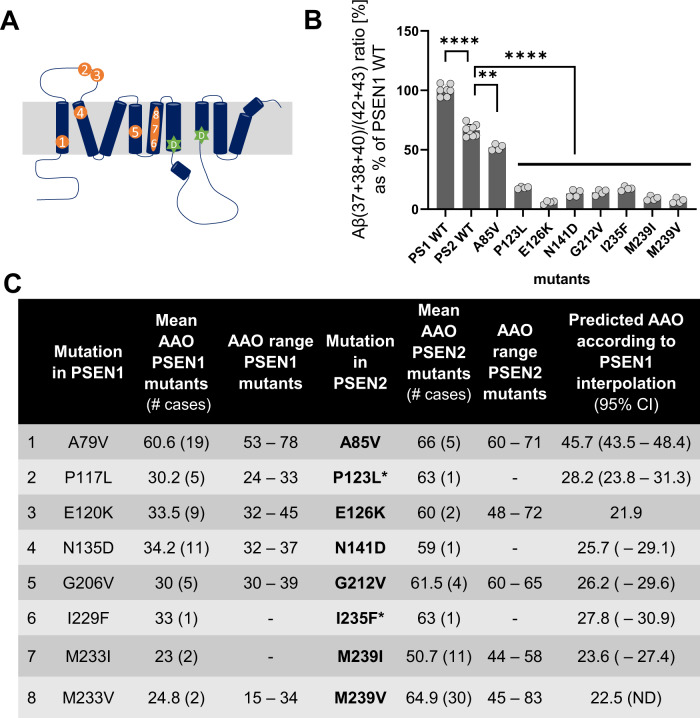
Aβ profiles generated by PSEN2-GSECs estimate the AAO of sister *PSEN1* variants. **A** Schematic representation of the localisation of the tested PSEN mutations on the primary structure of PSEN1/2. **B** Efficiency of the 4th GSEC turnover quantified by the Aβ (37 + 38 + 40) / (42 + 43) ratio. One-way ANOVA followed by Dunnett’s post-hoc test with comparison to wild type was used to determine statistical significance *p* < 0.05; **p < 0.01; ****p < 0.0001, (F(DFn, DFd): F(9, 37) = 147. Data are presented as mean ± SD of *N* ≥ 4 independent experiments. The corresponding PSEN2 Aβ profiles are shown in Fig. S4C. **C** Table presents the estimated AAO for the indicated FAD-linked PSEN1 mutations. The 95% CIs of each AAO prediction are ‘lower limit’ and ‘upper limit’. *indicates mutations with unclear pathogenicity (https://www.alzforum.org/mutations/psen-2).

In addition to PSEN1, PSEN2 mutations are implicated in FAD pathogenesis. PSEN1 is highly homologous with PSEN2, however, the activities and subcellular localisations of different type of GSECs differ significantly, with PSEN2-type complexes generating more longer Aβ peptides [40] mainly in the endosomal compartment [41]. Intriguingly, although PSEN2-type GSECs generate more amylogenic Aβ peptides relative to PSEN1-type ones, pathogenic variants in *PSEN2* are associated with later AAOs. The latter can be clearly appreciated when assessing the clinical phenotypes of 8 particular mutations affecting the same residue and position in both PSEN1 and PSEN2 catalytic subunits. The pathogenic *PSEN1* A79V, P117L, E120K, N135D, G206V, I229F, M233I and M233V mutations (Fig. 5A) cause FAD with AAO varying from 23 to 60 years, while carriers of the twin *PSEN2* mutations present AAO in the fifth or sixth decade (Fig. 5C), and the pathogenicity of two of the *PSEN2* variants (P123L and I235F) is unclear (www.alzforum.org/mutations/psen-2).

To gain insights into the biochemical aspects of this discrepancy, we analysed Aβ profiles generated by the 8 mutant PSEN2/GSECs (Fig. 5B). We reasoned that if similar mechanisms underlie PSEN1 and PSEN2 pathogenic effects, the Aβ profiles generated by the twin PSEN2 variants would estimate AAOs similar to those observed for the PSEN1 variants. We thus calculated the respective PSEN2 mutant Aβ (37 + 38 + 40) / (42 + 43) ratios and interpolated them in the PSEN1 correlative data (shown in Fig. 2B). Biochemical analysis of the PSEN2 Aβ profiles (Fig. 5C) estimated similar AAOs (in 5/8 cases) to the clinical onsets of the corresponding PSEN1 variants. Strikingly, the analysis of the three PSEN2 mutations that did not match the AAOs in PSEN1 carriers (A79V, E126K and N141D) predicted earlier onsets. These data suggest that similar pathogenic mechanisms operate in PSEN1 and PSEN2 variants and support the notion that lower contribution of PSEN2 type-GSECs to brain APP metabolism [42, 43] explains the “delayed” clinical phenotypes.

## Discussion

Whether FAD-linked mutation-driven changes in Aβ profiles correlate with AAO is of fundamental importance for basic and clinical research as well as for therapeutic development. Here, we investigated this central aspect of AD pathophysiology while considering the full spectrum Aβ peptides generated by a large number of pathogenic GSECs. This contrasts with previous studies focused solely on changes in Aβ42 and/or Aβ40 production [17, 18, 22–24]. Specifically, we analysed the composition of Aβ profiles generated by 25 FAD-linked PSEN1 mutant GSECs associated with a wide range of AAOs and applied hypothesis- as well as data-driven approaches to determine potential relationships between alterations in Aβ production and AAO.

Our mechanism-based approach demonstrates a remarkable linear correlation between the Aβ (37 + 38 + 40) / (42 + 43) ratio, reporting on GSEC processivity, and AAO. The significant correlation proves that the previously observed linear relationship between the degree of E-S destabilization and AAO [29] holds true at the level of secreted Aβ profiles. This is noteworthy as it supports the pathogenic role of shifts towards production of longer and more amyloidogenic Aβs [44].

Previous analyses of the relationships between Aβ42 (absolute or relative to Aβ40 increments) and AAO are inconsistent, with studies showing no significant [17, 22, 24, 45] as well as robust correlations [18, 23, 46]. Therefore, we also assessed potential scenarios that could lead to elevations in Aβ42: mutation-driven shifts in the GSEC product line preference to favour the Aβ42 product line and/or impairments in the conversion of Aβ42 into Aβ38, and evaluated their relationships with AAO. However, these GSEC features (assessed by the Aβ (38 + 42) / (37 + 40 + 43) and Aβ 38/42 ratios, respectively) revealed relatively weak correlations with AAO.

We also investigated relative Aβ40 vs Aβ42 changes, arising from impaired processivity [25], and found a significant linear correlation between the Aβ 40/42 ratio and AAO. Of note, the analogous analysis using the Aβ 42/40 ratio revealed that the inclusion of very destabilizing mutations -linked to very high Aβ 42/40 ratio- compromises this correlation. The apparent incongruence may arise from a ‘denominator problem’ caused by the low Aβ40 levels. The use of the Aβ 40/42 ratio resolves the apparent incongruency, while the inclusion of other Aβ peptides (Aβ (38 + 42) / (37 + 40 + 43)) further improves the correlation with AAO.

Previous studies reporting inconsistent data for the AAO-Aβ 42/40 correlation were proposed as a challenge for the role of Aβ in AD pathogenesis and suggested that alternative disease mechanisms could be operating in FAD. Most importantly, Sun et al. reported a lack of correlation between the Aβ 42/40 ratio and the AAO (R^2^ = 0.038) [24] and postulated that FAD occurs through Aβ-independent mechanisms. We note however that mutations abrogating the generation of Aβ42 and Aβ40 peptides in this report (~30% of the tested variants) have been shown by us and others to generate both peptides in cellular context; as examples the PSEN1 T291P and V412I mutants analysed here (see also Table S1). These apparent contradictory findings may be related to the analysis of Aβ generation in detergent conditions (purified, detergent-solubilized GSEC) by Sun et al. Detergent extraction per se destabilizes GSEC shifting Aβ generation towards longer peptides (>Aβ42) [29]. Similar phenomena have been reported for G protein-coupled receptors [47]. Furthermore, the inclusion of Aβ 42/40 ratios calculated from GSECs with nearly ‘zero activity’ levels may have added uncertainty to the study (discussed in [48]).

Overall, the mechanism-driven analysis strongly supports the notion that increments in the generation of longer Aβ peptides not only determine pathogenicity, but largely define clinical onset. This novel observation provided the basis for the estimation of clinical onset from Aβ profiles.

We also pursued a data-driven PCA approach. The analysis shows that earlier AAOs mainly correlate with enhanced generation of Aβ42 or Aβ42 and Aβ43. In addition, the inverse relationship between Aβ40 and Aβ42 suggests that alterations in the GSEC product line preference (favouring the Aβ42 product line over the Aβ40) influence AAO. We note that several FAD-linked mutations in *PSEN1* and *APP* indeed promote the Aβ42 product line [25, 34]. Intriguingly, the PCA biplot differentiates a number of pathogenic mutations (L166R, L235P, G266S, R278T, L282R and A431E). These variants are characterised by relative increments in Aβ43. We noted that carriers of the L166R, L235P, G266S, R278T and A431E variants (differing the most) present atypical phenotypes. For instance, spastic paraparesis affects 45% of *PSEN1-*A431E [49] mutation carriers and is reported as an early feature of the *PSEN1*-G266S mutation [50]. When we investigated the relationship between the PC1/PC2 and AAO in a multivariate model, we observed a linear correlation. Hence, both hypothesis- and data-driven analyses strongly support a significant linear correlation between Aβ profiles and AAO. Furthermore, both analyses point at longer Aβ42 and Aβ43 peptides as key factors in pathogenesis and clinical onset.

We note that the simple cell line-based assays used here lack the complexity seen in the FAD affected brain, where both mutant and normal PSEN1 as well as PSEN2 contribute to GSEC activity. However, the observed strong correlations between mutation-driven alterations in Aβ profiles and AAO provides compelling evidence that this assay reports on intrinsic biochemical changes relevant to human disease. Of note, the Aβ 42/40 ratio determined in our system for the wild type PSEN1 cell line (Aβ 42/40 = 0.14) is consistent with the Aβ 42/40 ratio generated by non-AD neurons (derived from control iPSCs) cultured in 2D or 3D conditions (Fig. 3A in Arber et al. 2020 [51]). Furthermore, similar relative enrichments in Aβ42 (vs Aβ40) are reported in 2D and 3D patient-derived neurons for the Ins113T, Y115H and M139V [51]; though (as expected) the magnitudes of the changes in the heterozygous cultures are lower than in our ‘homozygous’ cells (Aβ 42/40 = 0.25, 0.40 and 0.36 for PSEN1 Ins113T, Y115H and M139V in this report versus Aβ 42/40 ratios in Fig. 3A in Arber et al. 2020 [51]). Whether the more complex, heterozygous Aβ profiles generated in FAD correlate with AD onset, allowing AAO estimation is certainly of great interest and warrants further investigations.

As the next step, we investigated whether Aβ profile analysis could be used to determine mutation pathogenicity and predict AAO. Here, we emphasize the importance of quantitative approaches to investigate the potential pathogenicity and severity of variants of uncertain significance as next generation genetic analyses (whole exome or genome sequencing) are increasingly being used in clinical settings. The strong, linear relationship between the Aβ (37 + 38 + 40) / (42 + 43) ratio and AAO encouraged us to test the predictive value of this correlation. Specifically, we used an interpolation approach for the estimation of AAO in carriers of variants of unclear pathogenicity, including a potential association with FTD, or for providing insights into the pathogenic nature of *PSEN1* mutations with atypical phenotypes.

The S132A substitution has only been reported in one family. The proband carried an *APOE* ε3/ε4 genotype and developed AD at age 59 [3]. Intriguingly, the S132A and wild type Aβ profiles were virtually identical, even in conditions that have proven to boost the destabilizing phenotypes of mildly pathogenic *PSEN1* variants. Therefore, the biochemical analysis suggests that this may be a sporadic phenocopy, with the family history related to *APOE* ε4. In support of this conclusion, data released by the UK Biobank while this manuscript was in revision (https://genebass.org/gene/ENSG00000080815/phenotype/icd_first_occurrence-131036-both_sexes--?resultIndex=gene-manhattan&resultLayout=small) demonstrate that the *PSEN1* S132A mutation is not associated with AD (*P* = 0.8). Collectively the data support the non-pathogenic nature of the *PSEN1* S132A mutation.

In contrast, all other likely pathogenic variants demonstrated decreased Aβ (37 + 38 + 40) / (42 + 43) ratio and an interpolation analysis assigned intrinsic AAOs within <6.2 years from the actual clinical onset for 6 out of the 9 tested cases (L113P, V142I, Y154N, G266C, L282P and T291P) and intrinsic AAO values that differ by ~8 and ~12 years for the V393F and P433S variants, respectively. Here, it is important to note the inherent error in the determination of clinical AAO given the limited number of cases (Fig. 4D) and the fact that clinical onset is an insidious process that depends on the observation and recollection of family members.

The results indicate that changes in the Aβ (37 + 38 + 40) / (42 + 43) ratio largely determine AAO, but also suggest that additional genetic and/or environmental factors may play a modulatory role. In the case of the V393F variant carrier, we speculate that the presence of an *APOE* ε2/ε3 genotype may explain the mismatch between the ‘intrinsic’ (biochemical) and the clinical AAO. The *APOE* ε2 allele has been found to lower AD risk and/or delay its onset [52–54]. Interestingly, genetic analysis of the largest FAD PSEN1 E280A pedigree has revealed that the *APOE* ε2 allele delays AAO by 8.2 years [39]. Such an *APOE* ε2 protective behaviour would fit with the determined here intrinsic AAO for the PSEN1 V393F mutant (61 y vs. 52.7 y for reported and estimated AAOs). These observations suggest that pathological Aβ accumulation in FAD is being influenced by production as well as clearance.

The mismatch between the biochemical and clinical data for the P433S mutant brings to the discussion an important aspect of FAD pathogenesis. We have previously shown that strongly destabilizing PSEN1 mutations (such as R278I and L435F [27]) exert inhibitory actions on the global GSEC endopeptidase activity and have an apparent delay in clinical onset [29]. These observations propose that the extreme inactivating nature of these substitutions silences the disease allele and thus counteracts the inherent pathogenic effects of the mutation. The delay in clinical relative to intrinsic AAO for the P433S mutant supports this view. Here, it is also worth noting the similarity with the PSEN2-type GSEC, which generates Aβ profiles enriched in longer Aβs but a lower contribution to the metabolism of APP in brain, relative to PSEN1, may delay clinical onset.

The ‘silencing effect’ exerted by extremely inactivating PSEN1 variants, not only argues against a simple GSEC loss-of-function mechanism, but also supports the selective targeting of the pathogenic allele as a potential therapeutic approach in FAD. In this regard, antisense oligonucleotides offer nowadays hope for CNS disorders and the data derived from these pathogenic and silencing *PSEN1* mutations offer support for gene silencing therapy. Another key insight derived from these specific cases concerns the role of Aβ43-enriched profiles in AD pathogenesis: extremely inactivating FAD-linked *PSEN1* variants predominantly produce Aβ43 at very low levels [27–29, 55, 56] yet they cause FAD. As a note of caution, the production of even longer Aβ (Aβ45/Aβ46) species (that escape current detection methods) cannot be excluded.

Finally, two mutations in PSEN1 previously -and contentiously- associated with a FTD phenotype (L113P and V412I) were also tested. The L113P variant has been associated with autosomal dominant inheritance in five members of the same family, with a behavioural presentation reported for the three individuals with clinical data available, that was sufficient to fulfil criteria for FTD [57–59]. However, no consensus has been reached and two potential scenarios have been discussed: the mutation causes FTD or a frontal variant of FAD [60]. The analysis of the Aβ profiles indicates a pathogenic nature for L113P mutation and supports the FAD phenotype. Conversely, the association of V412I mutation with autosomal dominant FTD [61] has been challenged by the predicted variant’s penetrance: ‘most likely benign’ [62] – and our analysis – showing a lack of alterations in the V412I Aβ profile- demonstrates that this substitution does not share a common mechanism with other FAD variants and thus does not support a pathogenic nature.

What is the minimal required change in the molecular composition of Aβ profiles to initiate or delay (in therapeutic settings) AAO? This is a fundamental question that awaits further investigations. Nevertheless, our analyses provide insights into the composition of pathogenic Aβ cocktails and open avenues to investigate the bases of Aβ toxicity.

In conclusion, our studies not only provide fundamental insights but also offer a potentially valuable assay for clinical, genetic and therapeutic research. They set the conditions for the biochemical assessment of *PSEN1* mutation pathogenicity and AAO, which could be valuable for clinical genetic counselling, especially when considering that a substantial number of *PSEN1* mutations occur de novo [63]. Of significance, the utility of Aβ profiles to predict AAO has the potential to improve clinical and therapeutic design. Furthermore, determination of the intrinsic AAO in carriers of FAD-linked mutations may identify patients with a mismatch between clinical and biochemically estimated AAOs and point to potential genetic modulators of AAO. Last but not least, our data support the use of GSEC-targeting molecules securing GSEC-APP/Aβ_n_ interactions, and consequently shifting Aβ profiles towards short and less amyloidogenic peptides, as promising therapeutics in FAD and broadly in AD.

## Materials and methods

### Antibodies and reagents

The following antibodies were used in western blot analysis: anti-human PSEN1-CTF (MAB5232) and anti-human PSEN1-NTF (MAB1563) purchased from Merck Millipore; anti-human PSEN2-CTF (EP1515Y) purchased from Abcam; rabbit anti-PEN2 (B126) and mouse anti-NCSTN (9C3) kindly provided by Prof. Wim Annaert. Horse radish peroxidase (HRP)-conjugated anti-mouse (#1721011) and anti-rabbit IgG (#1721019) purchased from Bio-Rad and anti-rat IgG (#61-9520) purchased from Thermo Fisher. Antibodies used in the MesoScale Discovery (MSD) multispot Aβ ELISA were obtained through collaboration with Janssen Pharmaceutica NV (Beerse, Belgium). The MSD ELISA capture antibodies were JRD/Aβ37/3 for Aβ37, JRF AB038 for Aβ38, JRF/cAb40/28 for Aβ40, JRF/cAb42/26 for Aβ42, and the detection antibody was JRF/AbN/25 raised against the N terminus of Aβ. The antibodies used in the Aβ43 ELISA were anti-Aβ43 rabbit IgG (capture antibody) and anti-Aβ (N) (82E1) mouse IgG Fab’ (detection antibody), both supplied with the ELISA kit (IBL).

### Generation of wild type and mutant PSEN cell lines

In order to generate stable cell lines, *Psen1*^−/−^*Psen2*^−/−^ MEFs [64] were transduced with retroviruses, carrying pMSCVpuro plasmids encoding respective human wild type or mutant (A79V, V89L, F105C, Ins113T (intron4), Y115H, M139T, M139V, S132A, I143F, I143T, L166P, L166R, L171P, M233I, M233V, L235P, L235V, A246E, G266S, R269H, R278T, E280A, L282R, L381F, G384A, A431E, control variants: R35Q and E318G and novel/unclear pathogenic variants: L113P, V142I, Y154N, G266C, L282P, T291P, V393F, V412I and P433S) PSEN1s or mutant (A85V, P123L, E126K, N141D, G212V, I235F, M239I, M239V) PSEN2s, using a replication-defective recombinant retroviral expression system (Clontech). Non-pathogenic variants were selected by filtering for *PSEN1* missense changes found three or more times in the population. Accordingly, the selected R35Q and E318G substitutions are unlikely to be pathogenic, otherwise they would be commonly found in FAD patients. Of note, although the E318G variant has been associated with increased AD risk, recent studies failed to stablish an association with the disease. In line with these findings, our biochemical and γ-secretase thermoactivity data support the non-pathogenic character of the E318G mutation. To produce the retroviruses, HEK293T17 cells were co-transfected with pMSCVpuro wild type or mutant human PSEN1 encoding plasmids and a packaging vector [25]. Viral particles were collected 48 h post-transfection, filtered (0.45 µm pore size filter) and immediately used to transduce *Psen1*^−/−^*Psen2*^−/−^ MEFs cultured in Dulbecco’s Modified Eagle’s Medium (DMEM)/F-12 (Thermo Fisher Scientific) supplemented with 10% fetal bovine serum (FBS) (Sigma–Aldrich). Clones stably expressing target protein were selected with 5 μg/ml puromycin (Sigma–Aldrich). After three passages, puromycin concentration was reduced to 3 µg/ml.

### Western blotting

To confirm PSEN1 and PSEN2 expression and reconstitution of mature, active GSEC complexes, membranes were prepared and then solubilized in 1% CHAPSO, 28 mM PIPES pH 7.4, 210 mM NaCl, 280 mM sucrose, 1.5 mM EGTA pH 8 and 1x complete protein inhibitor mix (Roche). The protein samples were resolved on 4–12% Bis-Tris NuPAGE gels (ThermoScientific) and transferred to nitrocellulose membranes. Western blot analysis using the indicated antibodies, Western Lightning Plus-ECL Enhanced Chemiluminescence Substrate (Perkin Elmer) and Fuji imager was performed.

### Expression of APP_C99_ in MEF cell lines

For cell-based activity assays, respective MEF cell lines were transduced with recombinant adenoviruses carrying plasmids encoding human APP_C99_, as described previously [25, 38]. The adenoviral vectors encoded also green fluorescence protein (GFP), expressed from an independent promoter, allowing for control of the transduction efficiency. Briefly, cells were plated at the density of 25,000 cells/well into 48-well plates and 16 h later transduced with recombinant adenoviruses Ad5/CMV-APP. 7 h post-transduction the medium was changed to low-serum medium (DMEM/F-12 medium containing 0.2% FBS). After 24 h incubation at 37 °C or 42 °C (for thermoactivity assays), the conditioned medium was collected for Aβ analysis [38].

### Aβ peptide quantification in conditioned medium by ELISA

To quantify the concentration of Aβ37, Aβ38, Aβ40 and Aβ42 peptides, Multi-Spot 96-well MSD ELISA plates coated with anti-Aβ37, Aβ38, Aβ40 and Aβ42 antibodies were used. Non-specific protein binding to the plates was blocked with 150 μl/well blocking buffer (PBS supplemented with 0.1% casein) for 2 h at room temperature (while shaking at 600 rpm). 25 µl of SULFO-TAG JRF/AbN/25 detection antibody diluted in blocking buffer was mixed with 25 μl of standards (synthetic human Aβ1–37, Aβ1–38, Aβ1–40, and Aβ1–42 peptides at known concentrations) or 25 µl analysed samples, both diluted in blocking buffer, and the mix (50 μl/well) was loaded on the plate. After overnight incubation at 4 °C, the plates were rinsed 5 times with washing buffer and the signals were developed by the addition of 150 μl/well of the 2x MSD Read Buffer T (Tris-based buffer containing tripropylamine). The signals were read on a Sector Imager 6000 (Meso Scale Discovery). To quantify the concentration of Aβ43 peptides, conditioned medium samples were loaded on the ELISA plates coated with anti-human Aβ43 rabbit IgG, supplied with the human Amyloid β (1–43) (FL) assay kit (IBL), and Aβ43 peptide levels were measured following the supplier’s protocol.

### Data-driven analysis

Analysis was performed using R (v. 4.0.4) from raw ELISA data. For each mutant cell line, mean values for each Aβ specie were calculated and normalised to total Aβ so that 100% (Aβ_total_) = Aβ37 + Aβ38 + Aβ40 + Aβ42 + Aβ43. The data was centred and scaled by subtracting the mean-value of each feature and dividing by the corresponding standard deviation. Principle component analysis (PCA) was performed and the first two principle components (PC1/PC2) were selected for further analysis. PC1/PC2 explain 69.6% of all variation in the data. PCA biplot was generated using AMR package (v. 1.7.1). A multivariate linear model was used to describe AAO as a function of PC1 and PC2, and data visualised using plotly package (v. 4.9.3).

### Statistical analysis

All statistical analyses were performed using the GraphPad Prism 8, R v4.1.0 or 4.0.4 and R Studio software. One-way ANOVA with Dunnett’s post hoc test was used to test the significance of the changes between groups unless indicated otherwise. *P* < 0.05 was used as a pre-determined threshold for statistical significance. Linear regression was used to find the best-fit value of the slope and intercept (Y = intercept + slope*X), describing a linear relationship between Y (Aβ ratios) and X (AAO), and determine R^2^ (goodness of fit) and *P* values. Linear interpolation was used for assigning an X value to a given Y. All statistical analyses are described in the corresponding figure legends.

## Supplementary information

Supplementary data
