# Supplementary material for: Aβ profiles generated by Alzheimer’s disease causing PSEN1 variants determine the pathogenicity of the mutation and predict age at disease onset
Source: Mol Psychiatry. 2022 Apr 1;27(6):2821–32. doi: 10.1038/s41380-022-01518-6 (PMC9156411; doi:10.1038/s41380-022-01518-6)
Supplement: Supplementary file 1 — Supplementary data [file 41380_2022_1518_MOESM1_ESM.docx]

**Supplementary table legends**

**Table S1**

| **FAD-linked PSEN1 mutation** | **Effects of the mutations on Aβ generation in cell-based assays** |
| --- | --- |
| S212Y | Increased Aβ42 and Aβ40 levels (Ringman *et al*. 2011) |
| Q223R | Increased Aβ42 levels (Li *et al*. 2016) |
| I238M | Increased Aβ42 and Aβ40 levels (Ting *et al.* 2014) |
| K239N | Increased Aβ42 levels, unchanged Aβ40 levels (Sarroca *et al*. 2016) |
| L271V | Increased or unchanged Aβ42 levels, unchanged Aβ40 levels (Dumanchin *et al*. 2006 and Kwok *et al*. 2003) |
| T291P | Increased Aβ42 and Aβ40 levels (Dumanchin *et al*. 2006) |

**Supplementary table S1. Cell-free (detergent) and cellular data on the activity of mutant GSECs.**

The table lists PSEN1 mutations that yielded inactive GSECs, according to cell-free assays in [1](Sun et al. 2017). All these mutant GSECs presented rather unaltered global activity in cell-based studies [2–7](Ringman et al. 2011; Li et al. 2016; Ting et al. 2014; Sarroca et al. 2016; Dumanchin et al. 2006; Kwok et al. 2003).

**Table S2**

| **Mutation**  **PSEN1** | **Aβ37 [%]** | **Aβ38 [%]** | **Aβ40 [%]** | **Aβ42 [%]** | **Aβ43 [%]** | **N** |
| --- | --- | --- | --- | --- | --- | --- |
| **A79V** | 2.53±0.53 | 15.85±0.96 | 69.46±4.14 | 11.84±4.49 | 0.32±0.05 | 6 |
| **V89L** | 2.81±1.04 | 11.74±3.93 | 70.49±2.96 | 14.66±3.33 | 0.3±0.04 | 4 |
| **F105C** | 5.79±0.27 | 10.07±0.79 | 74.31±0.84 | 9.83±0.6 | 0±0 | 4 |
| **L113P** | 11.33±5.55 | 10.76±3.37 | 48.39±14.48 | 29.51±9.06 | 0±0 | 4 |
| **Ins113T** | 2.73±0.33 | 6.31±1.4 | 72.31±1.86 | 17.78±3.13 | 0.87±0.16 | 4 |
| **Y115H** | 6.91±3.5 | 8.43±1.53 | 59.37±4.35 | 23.63±5.41 | 1.67±0.9 | 5 |
| **S132A** | 4.98±1.2 | 13.58±1.55 | 72.2±2.46 | 9.21±3.21 | 0.03±0.07 | 10 |
| **M139T** | 8.11±7.9 | 10.84±2.84 | 62.25±8.48 | 18.21±4.18 | 0.6±0.5 | 5 |
| **M139V** | 5.94±3.82 | 10.59±2.02 | 60.64±3.9 | 22.12±1.82 | 0.71±0.26 | 5 |
| **V142I** | 4.28±0.22 | 11.73±0.46 | 66.48±5.11 | 17.51±4.93 | 0±0 | 4 |
| **I143F** | 1.88±0.84 | 22.06±1.21 | 61.85±5.02 | 14.07±3.36 | 0.14±0.07 | 4 |
| **I143T** | 6.02±0.64 | 29.39±1.24 | 41.45±1.95 | 23.01±0.7 | 0.13±0.01 | 4 |
| **Y154N** | 2.30±1.49 | 6.14±1.72 | 69.62±5.67 | 15.42±3.79 | 6.52±2.08 | 5 |
| **L166P** | 2.76±1.5 | 14.12±3.04 | 23.14±3.53 | 55.58±8.64 | 4.4±1.79 | 5 |
| **L166R** | 3.2±2.63 | 2.55±0.39 | 64.42±2.91 | 24.55±2.99 | 5.29±0.8 | 4 |
| **L171P** | 3.64±0.46 | 4.28±0.34 | 62.65±6.84 | 29.44±7.55 | 0±0 | 4 |
| **M233I** | 5.69±4.33 | 12.06±0.84 | 39.45±3.18 | 42.25±6.03 | 0.55±0.28 | 4 |
| **M233V** | 4.11±2.58 | 18.26±3.48 | 31.17±4.84 | 46.02±8.51 | 0.43±0.28 | 4 |
| **L235P** | 4.32±8.36 | 3.92±2.85 | 65.01±8.44 | 24.93±3.69 | 1.82±1.29 | 5 |
| **L235V** | 10.35±11.35 | 9.99±1.39 | 65.69±9.69 | 12.77±3.13 | 1.21±0.78 | 5 |
| **A246E** | 4.13±1.41 | 11.08±1.08 | 68.11±1.79 | 16.37±3.7 | 0.31±0.12 | 3 |
| **G266C** | 4.87±2.99 | 3.69±0.63 | 76.83±2.13 | 14.61±1.47 | 0±0 | 4 |
| **G266S** | 2.37±1.39 | 1.74±0.16 | 67.78±0.35 | 18.86±1.2 | 9.26±0.77 | 4 |
| **R269H** | 2.29±0.78 | 7.68±1.81 | 77.29±2.46 | 12.51±2.66 | 0.24±0.12 | 5 |
| **R278T** | 1.1±1.29 | 6.56±0.67 | 63.69±3.96 | 24.96±2.52 | 3.69±0.5 | 4 |
| **E280A** | 2.34±0.76 | 10.67±0.65 | 71.5±5.91 | 14.78±6.5 | 0.71±0.32 | 6 |
| **L282P** | 1.58±0.23 | 3.26±0.25 | 77.19±5.03 | 16.66±5.15 | 1.3±0.12 | 4 |
| **L282R** | 1.32±0.61 | 3.96±0.3 | 79.66±3.63 | 13.53±4.08 | 1.53±0.13 | 4 |
| **T291P** | 1.58±1.41 | 12.2±0.36 | 59.94±3.51 | 21±5.39 | 5.28±0.76 | 4 |
| **L381F** | 6.2±4.4 | 13.03±3.23 | 44.03±6.52 | 24.64±4.31 | 12.09±3.03 | 5 |
| **G384A** | 0±0 | 15.41±0.53 | 62.25±0.88 | 22.35±0.94 | 0±0 | 4 |
| **V393F** | 2.8±1.23 | 7.34±0.66 | 74.38±4.21 | 14.9±4.54 | 0.58±0.08 | 4 |
| **V412I** | 5.7±0.42 | 17.29±1.86 | 67.77±2.78 | 9.03±1.56 | 0.21±0.03 | 4 |
| **A431E** | 2.18±2.27 | 3.76±2.65 | 72.32±5.78 | 13.89±2.96 | 7.85±3.62 | 6 |
| **P433S** | 0±0 | 3.69±1.05 | 43.55±6.48 | 18.57±6.63 | 34.18±1.57 | 4 |
|  |  |  |  |  |  |  |
| **R35Q** | 3.37±0.88 | 11.44±0.42 | 74.84±4.82 | 10.28±3.79 | 0.07±0.11 | 6 |
| **E318G** | 3.32±1 | 11.7±0.94 | 73.86±5 | 11.06±3.28 | 0.06±0.09 | 6 |

**Supplementary table S2. Average Aβ_n_ production for PSEN1 GSECs.** Data related to Figures 1B and 4A. The table presents the average Aβ_n_ production as percentage of total Aβ profiles ± SD. Data for WT cell line: 5.8 ± 3.4; 13.2 ± 3.3; 70.4 ± 6.8; 10.4 ± 3.5 and 0.19 ± 0.2 for Aβ37, Aβ38, Aβ40, Aβ42 and Aβ43 peptides; respectively.

**Table S3**

| **Genotype** | **Aβ37 [%]** | **Aβ38 [%]** | **Aβ40 [%]** | **Aβ42 [%]** | **Aβ43 [%]** | **N** |
| --- | --- | --- | --- | --- | --- | --- |
| PSEN1 WT | 4.28 ± 0.96 | 10.55 ± 0.93 | 79.10 ± 2.39 | 6.02 ± 0.68 | 0.05 ± 0.04 | 7 |
| PSEN2 WT | 4.76 ± 2.32 | 5.14 ± 0.51 | 80.94 ± 4.07 | 8.98 ± 1.30 | 0.17 ± 0.16 | 8 |
| PSEN2 A85V | 3.77 ± 2.67 | 4.74 ± 0.93 | 80.15 ± 4.74 | 11.22 ± 1.06 | 0.12 ± 0.12 | 4 |
| PSEN2 P123L | 3.05 ± 0.75 | 3.26 ± 0.24 | 66.80 ± 3.35 | 26.59 ± 2.49 | 0.29 ± 0.14 | 4 |
| PSEN2 E126K | 2.13 ± 1.03 | 2.01 ± 0.70 | 41.83 ± 5.09 | 53.35 ± 6.43 | 0.68 ± 0.21 | 4 |
| PSEN2 N141D | 5.64 ± 4.94 | 4.80 ± 2.14 | 55.58 ± 4.41 | 33.57 ± 4.65 | 0.40 ± 0.12 | 4 |
| PSEN2 G212V | 2.11 ± 1.07 | 2.91 ± 0.77 | 62.83 ± 3.54 | 32.03 ± 3.35 | 0.11 ± 0.09 | 4 |
| PSEN2 I235F | 3.18 ± 0.78 | 3.15 ± 0.29 | 65.92 ± 2.26 | 27.67 ± 1.71 | 0.09 ± 0.10 | 4 |
| PSEN2 M239I | 4.59 ± 2.49 | 7.28 ± 0.40 | 45.73 ± 3.44 | 42.28 ± 3.18 | 0.12 ± 0.09 | 4 |
| PSEN2 M239V | 8.08 ± 3.55 | 10.33 ± 1.19 | 32.61 ± 5.12 | 48.98 ± 5.32 | 0.003 ± 0.01 | 4 |

**Supplementary table S3. Average Aβ_n_ production for PSEN2 GSECs.**

The table presents the average Aβ_n_ production as percentage of total Aβ profiles.

**Supplementary figure legends**

**Figure S1. Rescue of GSEC expression in wild type/mutant PSEN1 MEF cell lines**

Representative SDS-PAGE/western blot analysis of CHAPSO-solubilized membrane proteins from *Psen1^-/-^/Psen2^-/-^* MEF cell lines stably expressing wild type or mutant human PSEN1 or PSEN2 subunits. Rescue of the active GSEC complex is evidenced by the presence of mature, glycosylated NCSTN and the PSEN1 endoproteolysed C-terminal fragment (PSEN1-CTF)). Arrowheads indicate the position of molecular weight markers. The R278I mutant was included as a reference FAD mutant displaying strong deleterious effect PSEN1/GSEC autoactivation and leading to reduced levels of PSEN1 CTF and accumulation of full length PSEN1 (FL PSEN1). The novel, inactivating P433S mutant also displays accumulation of FL PSEN1, but to a lower extent.

**Figure S2. Correlative analyses investigating potential relationships between increased Aβ42 with AAO**

Correlative analysis between AAO and (**A**) Aβ (38+42)/(37+40+43) ratio (product line preference), (**B**) Aβ 38/42 (efficiency of the conversion of Aβ42 into Aβ38), **(C)** Aβ 40/42 and (**D**) Aβ 42/40. ROUT test (Q=1) marks the L166P, M233I and M233V mutations (1, 2 and 3, respectively) as outliers. In all panels, the 95% confidence interval (light grey surface) and correlation coefficient (R^2^) are shown. The error bars present SD and range for Aβ ratio and AAO, respectively.

**Figure S3. PCA analysis of Aβ profiles and proportion of the total variance explained by new PC1-5 variables.**

The bar graph present contribution of particular Aβ species to the PC components.

**Figure S4. GSEC activity data on selected mutant PSEN1/2 GSEC proteases**

**(A)** Total Aβ production (defined as the sum of the measured peptides) of the P433S inactivating PSEN1 mutant was normalized to the levels of the corresponding wild type (from each experiment). **(B)** Secreted Aβ profiles normalized to total Aβ levels (Aβ 37+38+40+42+43) in MEF cell-based activity assays with wild type or mutant GSECs subjected to temperature challenge (42°C). (**C**) Secreted Aβ profiles generated by mutant PSEN2 GSECs normalized to total Aβ (defined as the sum of measure Aβ peptides). Data are represented as mean ± SD, N ≥ 4.

**References**

1. Sun L, Zhou R, Yang G, Shi Y. Analysis of 138 pathogenic mutations in presenilin-1 on the in vitro production of Aβ42 and Aβ40 peptides by γ-secretase. Proc Natl Acad Sci U S A. 2017;114:E476–E485.

2. Kwok JBJ, Halliday GM, Brooks WS, Dolios G, Laudon H, Murayama O, et al. Presenilin-1 mutation L271V results in altered exon 8 splicing and Alzheimer’s disease with non-cored plaques and no neuritic dystrophy. J Biol Chem. 2003;278:6748–6754.

3. Ringman JM, Gylys KH, Medina LD, Fox M, Kepe V, Flores DL, et al. Biochemical, neuropathological, and neuroimaging characteristics of early-onset Alzheimer’s disease due to a novel PSEN1 mutation. Neurosci Lett. 2011;487:287–292.

4. Ting SKS, Benzinger T, Kepe V, Fagan A, Coppola G, Porter V, et al. A novel PSEN1 mutation (I238M) associated with early-onset Alzheimer’s disease in an African-American woman. J Alzheimer’s Dis. 2014;40:271–275.

5. Li N, Liu K, Qiu Y, Ren Z, Dai R, Deng Y, et al. Effect of presenilin mutations on APP cleavage; Insights into the pathogenesis of FAD. Front Aging Neurosci. 2016;8.

6. Sarroca S, Molina-Martínez P, Aresté C, Etzrodt M, García de Frutos P, Gasa R, et al. Preservation of cell-survival mechanisms by the presenilin-1 K239N mutation may cause its milder clinical phenotype. Neurobiol Aging. 2016;46:169–179.

7. Dumanchin C, Tournier I, Martin C, Didic M, Belliard S, Carlander B, et al. Biological effects of four PSEN1 gene mutations causing Alzheimer disease with spastic paraparesis and cotton wool plaques. Hum Mutat. 2006;27:1063.
